# Looking Back After the First Year of the COVID-19 Pandemic: Parents’ View on Screen Media Use, Psychopathology, and Psychological Burden in a Clinical Sample of Children and Adolescents

**DOI:** 10.3390/healthcare13162026

**Published:** 2025-08-17

**Authors:** Anna Maria Werling, Susanne Walitza, Renate Drechsler

**Affiliations:** 1 Child and Adolescent Psychiatry and Psychotherapy, Centre for Child and Adolescent Psychiatric Research, University Hospital of Psychiatry Zurich, University of Zurich, 8032 Zurich, Switzerland; 2 Neuroscience Center Zurich, University of Zurich and ETH Zurich, 8057 Zurich, Switzerland

**Keywords:** problematic use of the internet, screen media use, COVID-19, pandemic, child and adolescent psychiatry, ADHD, depression

## Abstract

**Objectives.** The aim of this study was to examine screen media use and the development and alteration of pre-existing mental health problems over the first year of the COVID-19 pandemic in a clinical sample of children and adolescents. **Methods.** A survey was conducted with over 650 parents of patients referred to child and adolescent psychiatry. **Results.** A worsening of the main mental health problem during the first year of the pandemic was reported more often (38%) by parents than an improvement (25%), a tendency even more pronounced for comorbid/secondary problems (worsening in 48%, improvement in 16% of cases). Girls in general, but especially between 14 and 18 years, more often showed a persistent deterioration of mental problems than boys. A negative impact of screen media use on their child’s mental health was reported most frequently by parents of a child affected by depression (50%), with acute crisis (52%), eating disorders (45%), and conduct disorder/aggression (40%). Patients with multiple/comorbid mental health problems presented significantly higher mean screen media times (5.53 h/day) than patients with a single mental problem (3.97 h/day), and their parents more frequently reported increased concerns about their child’s media use since the pandemic. Critical periods such as the lockdown or the “second wave” outbreak were characterized by higher media consumption in all patients, but with higher peaks in patients with multiple mental health problems. **Conclusions.** Particularly vulnerable patients, i.e., those with multiple mental problems and adolescent girls, have become more vulnerable in the course of the pandemic. From the perspective of most parents, screen media use has contributed to this development.

## 1. Introduction

The COVID-19 pandemic and its societal measures have profoundly impacted the mental health of children and adolescents [1,2,3,4,5,6,7]. Besides concerns about the virus infection [8], consequences of protective measures such as school closure, altered daily routines, isolation, and restrictions on leisure and sports activities have been associated with psychological stress and represented a risk to their mental well-being during this challenging time [6,9,10,11]. Another consequence was that all social and many leisure activities had to take place online and that screen media use dramatically increased, (e.g., [12,13]).

### 1.1. Impact of the Pandemic on Children’s and Adolescents’ Mental Health

Studies investigating the consequences of the pandemic on children’s and adolescents’ mental health have led to inconsistent results, which may be related to differences in the investigated time periods, populations, and strictness of measures taken by the respective national governments.

The psychological strain was not consistently high throughout the pandemic, due to the different “virus waves” (peaks of incidences), the alternation between stricter and more relaxed restrictions, and the uncertainty about future waves. Most research focused on the impact of the COVID-19 pandemic in earlier stages or on pre/post-lockdown comparisons [14,15,16,17,18,19].

Studies conducted shortly after the lockdown found that prevalence rates of internalizing disorders (with anxiety and depressive symptoms) increased or nearly doubled (i.e., [16,20]; see [21]) compared with pre-pandemic estimates, with older adolescents and girls being most affected ([22]; see [21]). Other studies, however, found similar levels of child well-being after the lockdown to those before the outbreak [23,24], or only small deteriorations [25]. The longer-term mental aftermath of the lockdown and the effect of repeated pandemic peaks have been less investigated (see [26] for the timing of publications), but longitudinal studies are beginning to emerge [27]. A majority of studies found an aggravation of mental problems such as anxiety and depression over time in children and adolescents and increased behavioral symptoms [28,29,30,31,32]. According to an international collaborative study [33], depressive symptoms in adolescents increased between 2020 and 2022 from 9.0 to 17.0%, with older adolescents and females being more at risk. Others reported that most children and adolescents from the general population showed a resilience or improvement of mental health during the years following the first lockdown, while a small group continuously presented mental problems ([34,35]; for a review see [27]).

Symptom changes in children and adolescents were not only influenced by time, but also by region [36], as the time frame of different waves, the strictness of protective measures, and the numbers and duration of school closures varied between countries. It has been shown that school closure had negative effects on children’s mental health [37,38]. In Switzerland, after the spring 2020 lockdown, (compulsory) schools were allowed to remain open during subsequent waves and overall measures were less strict compared to neighboring countries. Nevertheless, a dramatic rise in demands for treatment in child and adolescent psychiatry and in referrals to psychiatric emergency care was observed, although not during or directly after the lockdown, but from autumn 2020 (“second wave”) onwards, with a peak in winter 2021 [39,40,41]. Cases of depression and suicidal crises particularly increased. A comparable pattern was observed in Germany [42].

Only a small number of studies focused on clinical samples, i.e., patients with pre-existing psychopathology or newly referred to child and adolescent psychiatry. Adolescents with pre-existing mental health problems seemed most at risk to be negatively affected by the pandemic according to a large cooperative study [33], but several studies reported opposite results, with higher risks for healthier groups [43] and better outcomes in adolescents at risk [44]. While, in patients, a deterioration of pre-existing symptoms has been described [32], as well as a higher mental burden compared to non-clinical samples [45], several studies also reported a certain relief of stress. In a German psychiatric sample collected from autumn 2020 until spring 2021, a moderate to severe deterioration of mental problems was reported by 72% of parents and improvements by 12% [46]. Improved symptoms during the lockdown have been reported especially in subgroups with mental disorders, such as social or school anxiety, learning disorders, or autism spectrum disorder (ASD) [47,48,49,50] but also pre-existing depression [51]. Although the impact of the pandemic seemed to differ according to the various pre-existing psychopathological disorders, reported effects are not always consistent (e.g., [52]). The overall effects on ADHD and ASD were mixed, but mostly negative [53,54]. Eating disorders in adolescents have been found to increase during the pandemic [55]. The impact on pre-existing obsessive compulsive symptoms is unclear but seems rather associated with an exacerbation [56].

### 1.2. Impact of the Pandemic on Screen Media Use in Children and Adolescents

Studies reported a general increase in media time during the lockdown, followed by a partial decrease or a complete return to pre-COVID-19 levels after the easing of measures [18,43,57,58]. Results on the risks of high media use during the lockdown were mixed, with positive effects on coping with the pandemic [19,59,60,61], but increased risks for internet and video gaming addiction [62]. As for the pandemic’s impact on screen media use beyond the effects of the lockdown in spring 2020, most longer-term studies found increased risks and prevalence rates for the problematic use of the internet (PUI) in adolescent clinical and population samples ([63,64,65,66]; for definitions and models of PUI, see [67,68]), and an association between ill-being and social media use and ill-being and media addiction [69]. Media use varied considerably between different time periods of the pandemic, with higher media time during peak incidence periods [70]. Higher media times in autumn 2020 to spring 2021 were found to be associated with higher depressive symptoms, which was most pronounced in adolescent girls [71].

### 1.3. The Present Study

The present study complements other studies from our research team on the effects of the COVID-19 pandemic on children and adolescents with pre-existing mental vulnerabilities [17,18,19,39,40]. Our first survey with parents of children and adolescents referred to child and adolescent psychiatry, which matches the present study, was conducted directly after the lockdown in spring 2020 [17,18]. At the time, we found a significant increase in screen media use during the lockdown weeks in both genders, and a return to pre-lockdown screen media times after the easing of measures in females, while media use remained slightly increased in males. The aim of the present study was to assess the impact of the COVID-19 pandemic on the mental well-being and media use of clinically referred children and adolescents after the first year of the pandemic. We aimed to investigate whether screen media use had further increased or fluctuated during the pandemic and to which extent these changes were associated with mental health problems. We particularly focused on the parents’ view of changes in the severity of mental problems over time, on changes in screen media habits, and on possible associations between screen media use and well-being. We further wanted to examine which problems or disorders might have been exacerbated or improved during the first year of the pandemic and which subgroups of patients had been most affected. Finally, we were interested in the parents’ personal views when looking back at the first year of the pandemic.

## 2. Materials and Methods

### 2.1. Recruitment

Parents of patients aged between 10 and 18 years, who had been referred to outpatient clinics of Child and Adolescent Psychiatry and Psychotherapy (CAPS), University Hospital of Psychiatry Zurich, Switzerland, during the last three years were invited to participate in an anonymous online survey. The CAPS treats patients from the whole Canton of Zurich (1.5 million residents), including socioeconomically diverse locations with rural, urban, and industrial areas. The study link was sent by email, including comprehensive information on this study, to approximately N = 3400 parents of patients. The survey could be completed anonymously by the parent(s). The study was conducted in accordance with principles of the declaration of Helsinki and in agreement with the local Ethics committee.

### 2.2. Context

Data collection started on 12 May 2021, and was completed on 4 June 2021. In Switzerland, restrictions to contain the spread of the coronavirus were less severe compared to other countries: a complete lockdown with school closures lasted from 16 March to the end of April 2020. From 11 May 2020, onwards, the first schools re-opened and were allowed to stay open for the remaining time of the pandemic, which includes “second wave” virus peak incidences with the reintroduction of strict protective measures in autumn 2020/winter 2021 (the governmental restrictions and changes related to the different points in time referred to in the survey are summarized in Table S1).

### 2.3. Instruments

Media-use-related items were based on the parent version of the Screening Questionnaire for Problematic Use of the Internet (PUI-SQ Parents, [17,18]). The PUI-SQ Parents was developed by Werling and Drechsler for the routine screening of the problematic use of the internet/screen media and media-related problems in child and adolescent psychiatry. The questionnaire comprised the following subscales/domains: 1. Screen media time concern subscale: Parent’s concern about the amount of time spent on different digital devices and activities (five items); 2. Negative impact subscale: Impact of screen media use on everyday life and addiction-like behaviors (seven items); 3. Concern about digital risks and problems subscale (four items; the original subscale of 8 items was shortened by four items for the purpose of this study); 4. Leisure screen media time per day (six items). We added two new questions about COVID-19-related changes in digital media habits (2 items). Items from the PUI questionnaire were rated on 4-point Likert scales (from “not true” to “absolutely true”), except for leisure screen media time per day, which was rated on a 5-point Likert scale (no time at all and <1, 1–3, 4–6, and >6 h). For further analyses, the mean of each time range was used as an estimate of mean screen media time (0, 0.5, 2, 5, and 7 h). The two new questions were also rated on 5-point Likert scales. When not otherwise specified, questions had to be answered with reference to March/April 2021.

In addition, they was asked for (1) the main and secondary psychopathology (“main problem”, “secondary problem”) for which the child had been referred to the CAPS, with the possibility of choosing from preselected categories, and a free text field for specification (for problem categories, see Tables S2 and S3); (2) changes to the main and secondary problem during the pandemic; (3) changes in psychological burden, severity of symptoms, and screen media habits in the course of the pandemic; (4) retrospective estimations of the major psychological distresses for patients and parents during the pandemic.

Additionally, the present study used several items from the European collaboration study on the impact of COVID-19 on children and adolescents with pre-existing mental health problems (CRISIS) of the ECNP group (see [72]) to assess demographic characteristics, the financial situation of the family, and schooling.

### 2.4. Statistics

The main results were analyzed descriptively. Comparisons of categorical data were calculated using chi^2^ tests. Interval-scaled data were analyzed using *t*-tests and ANOVAs. For analyses of gender effects, patients with gender identity conditions were excluded due to their small number.

## 3. Results

### 3.1. Sample Description

In total, N = 1002 families took part in this study, resulting in a response rate of 33.8%. Responses from parents of a child younger or older than the indicated age range of 10 to 18 years or with missing data on media use and demographic information were excluded (N = 316). Finally, N = 686 parent ratings with complete data sets were analyzed, resulting in a total response rate of 33.8% (incomplete participations included) or 20.2% (complete participations only). A proportion of 90.3% of the respondents were mothers, including mothers by adoption and foster mothers, and 9.6% were fathers.

Of the total responses, 49.9% referred to male (N = 342), 46.8% to female (N = 321), and 3.4% to “other/diverse” patients (Table 1). The patients’ mean age was 13.5 y (SD = 2.81), with female patients being significantly older on average (13.9 y, SD = 2.70), due to the smaller number of younger and higher number of older female patients compared to males, which is characteristic of the distribution of gender in child and adolescent psychiatry. Patients were categorized into three age groups (8 to 10, 11 to 13, and 14 to 18 y). The oldest group accounted for more than half of the sample (54.1%, Table 1). Within these three age groups, age differences between male and female patients were not significant. N = 23 patients had received counseling or treatment related to gender dysphoria. These patients were older on average than male or female patients. Because of the small size (N = 23), this group was not included in gender comparisons.

In winter 2021, 64% had a regular school situation (in the classroom, at regular school times); 16% went to school on-site, but with a reduced frequency (e.g., only some days a week); 6% had received only online schooling, with teacher interaction during limited times; and 4% had only online schooling, at normal school hours. The majority of respondents (86.2%) indicated that their current financial situation was good (75.2%) or at least acceptable (10.9%); the financial situation of the remaining group was quite (10.1%) or very difficult (3.4%). Some reported that they (or their partner) had lost their jobs (2.2%) or were currently working short-time (5.3%) (reduced hours because of the pandemic with compensation). A proportion of 21.5% of the sample had also participated in the first survey directly after the lockdown [17,18].

### 3.2. Distribution of Psychopathological Problems

ADHD, autism spectrum disorders (ASDs), depression, acute crises, anxiety, and obsessive/compulsive disorders (OCDs) were among the most frequently reported main disorders. For subsequent analyses, all the main problem categories comprising fewer than N = 20 patients (i.e., learning disorder, Tic/Tourette, trauma, psychosomatic problems, social/family problems, psychosis/borderline, school absenteeism/mutism; Table S2) were subsumed under the category “other”. In the different problem categories, gender distribution was not balanced, which was to be expected according to the prevalence rates of these disorders, e.g., with significantly more male patients with ADHD or ASD and more female patients with depression or eating disorders.

A proportion of 42% of parents indicated that there was no additional problem. Younger patients (8–10 y) had secondary problems/comorbidities (43.4%) less often than older patients (11–13 y: 55.9%, 14–18 y: 53.3%). Male and female patients did not differ in the frequency of comorbid problems. Among the most frequently reported secondary problems were depression, OCD, school absenteeism/social withdrawal, anxiety, ADHD, problematic use of the internet/gaming, and eating disorders (for the distribution of comorbidities across main problems, see Table S2).

### 3.3. State of Mental Health Before and During the Pandemic

As patients had been treated over the last three years at the CAPS outpatient clinics, we asked parents to indicate whether the main problem had still been present during the first year of the pandemic. According to 64.4% of responses, the main problem existed before the outbreak as well as during the pandemic. In another 10.8% of cases, the problem already existed before the pandemic, but in a milder form. In a further 11.4% of cases, the problem, i.e., the reason for the referral to the CAPS, had been resolved or partially resolved before the pandemic (particularly in patients with main diagnoses of anxiety disorders (20.5%), OCD (21.6%), acute crises (20%), and eating disorders (16%)). In a further 7% of cases, the problem emerged during the pandemic. Among these newly emerged cases, diagnoses of depression, eating disorders, and acute crises/suicidality were particularly frequent. Among the secondary problems, 7.8% had newly appeared during the pandemic, with problematic use of the internet (PUI), depression, social withdrawal/school absenteeism, anxiety, and eating disorders as the most frequently reported disorders.

### 3.4. Impact of the Pandemic on the Severity of the Main and Secondary Mental Problems

To determine the impact of the pandemic on children’s mental health problems, parents were asked whether the severity of the main problem had changed during the first year of the pandemic.

In the total group, a deterioration of the main problem occurred more frequently (28.4%) than an improvement (20.4%), but problem severity remained stable in almost a third of cases (30.8%) (Table 2). About 20% of participants did not respond to this question, which was due to the number of patients who were remitted before or had become mentally ill after the outbreak of the pandemic. Younger patients tended towards an improvement rather than an aggravation of symptoms, while the opposite was found in the older groups. Significantly more girls than boys showed an aggravation of the main mental problem (males, 22.8%; females, 34.9%; chi^2^, 28.375; *p* < 0.001) (Table 2).

Changes in severity were also analyzed regarding differences between disorders/main mental health problems. Several disorders displayed balanced developments over the year of the pandemic, with relatively high stability and approximately equal numbers of deterioration and improvement (ADHD, ASD, gender dysphoria). Some others, by contrast, tended towards aggravation (depression, acute crises, eating disorders, OCD), and two disorders tended towards improvement (anxiety disorders, aggression/conduct disorder) (Table 3, percentages refer to cases with valid responses). As for the secondary mental health problem, almost half (48%) showed a deterioration and only 16% an improvement. The proportion of deterioration was particularly high among the secondary disorders of PUI, school absenteeism/withdrawal, eating disorders, and depression.

Except for comorbid learning disorders, which remained mostly stable, the negative impact of the pandemic seemed higher overall on secondary problems (deteriorated: 48.1%) than on the primary mental health problems (deteriorated: 37.7%) (Table 3).

### 3.5. Screen Media Use

**Figure 1:**
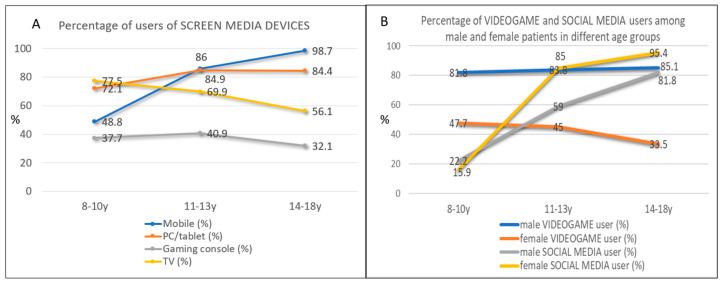
Percentage (%) in different age groups of screen media device users (**A**) and videogame and social media users (**B**).

In the total sample, the estimated mean screen media time during leisure activities was, according to parents, 4.81 h per day (Table 4). Total screen media time significantly increased with age (F = 98.292; *p* < 0.000 (Table 4, Tables S3 and S4)), from 2 ½ to almost 6 h per day (8–10 y: mean, 2.58 h; 11–13 y: mean, 4.52 h; 14–18 y: mean, 5.83 h). Sex effects of total screen media time (male/female) were not significant when controlled for age (F = 1062; *p* < 0.303). Screen media habits considerably changed with age (Figure 1A, Table 4 and Table S4): 51.2% of 8–10 year olds did not use smartphones, compared to 1.3% of 14–18 year olds, and only 22.5% of 8–10 year olds never watched TV, while among the 14–18 year olds, a majority of 67.9% was completely TV-abstinent.

**Figure 2:**
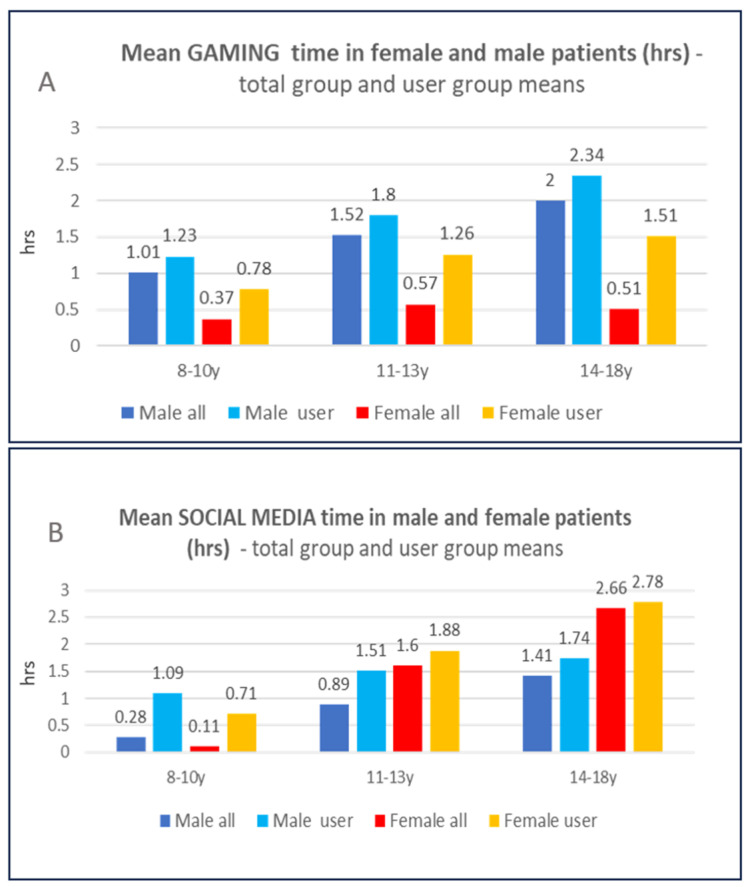
Mean video gaming time (**A**) and mean social media time (**B**) in female and male patients at different ages, in total groups and user-only groups.

In general, male patients had significantly higher video gaming times than females. A significant difference between male and female video gaming times remained when only users were included, at least in 14 to 18 year olds, with a mean daily gaming time in male users of 2.34 h and in female users of 1.51 h (Figure 2A, Table S5).

In general, most male patients used to play video games, starting in the youngest group by 81%, a percentage that slightly increased to 85.1% in the 14–18 y group. In female patients, less than half of the youngest age group used to play video games, a number that decreased to 33% in 14–18-year-olds (Figure 1A). However, female patients had significantly higher social media times, but only in the oldest age group (Figure 2B, Table S5). In 14- to 18-year-olds, over 80% of male and 95% of female patients used social media.

### 3.6. Association Between Mental Health and Screen Media Use

**Figure 3:**
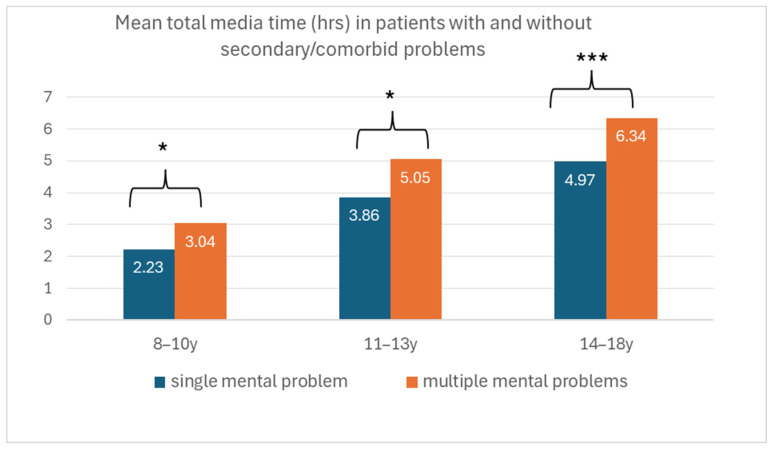
Estimated total screen media time (hours) in patients with and without secondary problems in different age groups (* *p* < 0.05; *** *p* < 0.001).

Patients with secondary/comorbid problems (N = 395, mean estimated total screen media time = 5.53 h, SD 3.66) had significantly higher total screen media times than patients with a single mental problem (N = 291, mean estimated total screen media time = 3.97 h, SD 2.49) (*p* < 0.001; Table S6), an effect that was significant across all age groups (Figure 3).

**Figure 4:**
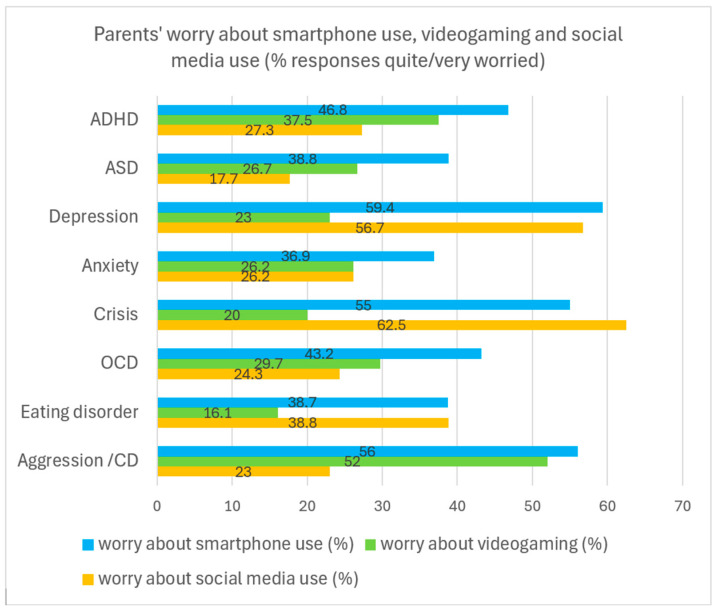
Parents’ responses (% quite/absolutely true) to questions on worrying about smartphone use, video gaming, and social media use in patients with different main problems. Numbers indicate the percentage of responses within the disorders. Example: Of all parents of a child with ADHD (=100%), 46.8% quite or absolutely agreed to being worried about their child’s smartphone use, 37.5% of parents agreed to being worried about video gaming, and 27.3% quite or absolutely agreed to being worried about social media use. Note. ASD = Autism Spectrum Disorder, OCD = Obsessive–Compulsive Disorder, CD = Conduct Disorder. The diagnostic groups are of different sizes.

As for problematic screen media use, a sizable proportion of parents were quite or very worried about the time spent by their child with their smartphone, on the internet, video gaming, and with social media (Table 5). Parents of a child with depression, in acute crises or with aggression/CD, were most often (about 55–60%) worried about their child’s smartphone use, with the two first ones being especially worried about their child’s excessive social media use, and the latter ones being worried about the time spent video gaming (Figure 4). Parents of a child with ADHD also frequently indicated worrying about the time spent with smartphones and video gaming.

**Figure 5:**
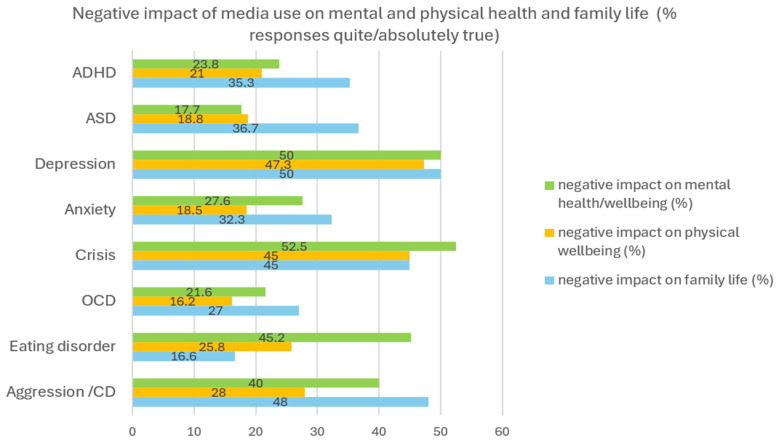
Negative impact of screen media use indicated by parents on mental health, physical health, and family life. Numbers indicate the percentage of responses within the disorders. Example: Of all parents of a child with ADHD (=100%), 23.8% quite or absolutely agreed with the statement that screen media use has a negative impact on their child’s mental health, 21% quite or absolutely agreed that it has a negative impact on their child’s physical health, and 35.3% quite or absolutely agreed that it has a negative impact on family life. The diagnostic groups are of different sizes. ASD = Autism Spectrum Disorder, OCD = Obsessive–Compulsive Disorder, CD = Conduct Disorder.

Parents were also asked about the negative effects of screen media use on their child’s health/well-being and about its consequences on everyday life. About half of parents with a child affected by depression (50%) or with acute crisis (52%) reported a negative impact of media use on their child’s mental health, followed by eating disorders (42%) and aggression (40%), which was more frequent than in patients with other disorders (average = 29.6%) (Figure 4). A negative impact of screen media use on physical well-being (e.g., on sleep) was also frequently reported by parents of children with depression (47.3%) and acute crises (45%). A negative impact of the child’s screen media use on family life was reported by almost a third of parents (response quite/absolutely true, 31%), but particularly frequently for patients with depression, crises, and aggression/CD (Figure 5). A proportion of 16.4% (N = 113) of parents reported being worried about their child being a victim of cyberbullying (cyberbullying: to harass, insult, or threaten someone on the internet or to publish false or unauthorized personal information) (quite/absolutely true) (Table 5). A proportion of 58.4% of this group were female patients, 38% were male, and 2.7% were diverse. The mean age of patients was 13.8 y (SD, 2.6 y). Parents were less frequently concerned that their child might be a cyberbullying perpetrator (6.5%). About half of these possible perpetrators were male (n = 23) and half were female (n = 22); the mean age was 13.6 y (SD 2.8 y). Cyberbullying as a perpetrator and victim was strongly correlated (r = 0.558, Spearman), showing that adolescents at risk of becoming a cyberbullying perpetrator have also been a victim or are at risk of becoming a victim.

### 3.7. Impact of the Pandemic on Screen Media Use

Parents were asked whether the pandemic had changed their view on their child’s screen media use: 30.5% reported being quite/much more worried today about their child’s screen media use than before the pandemic and 16.5% were somewhat more worried, while there was no change for the majority of parents (not/scarcely more worried: 52%). Parents of male or female children did not significantly differ in their responses, except for the youngest age group (8–10 y, quite/very true: male 24% vs. female 11%) (Figure S1), which may be explained by the low proportion of screen media users in young girls.

**Figure 6:**
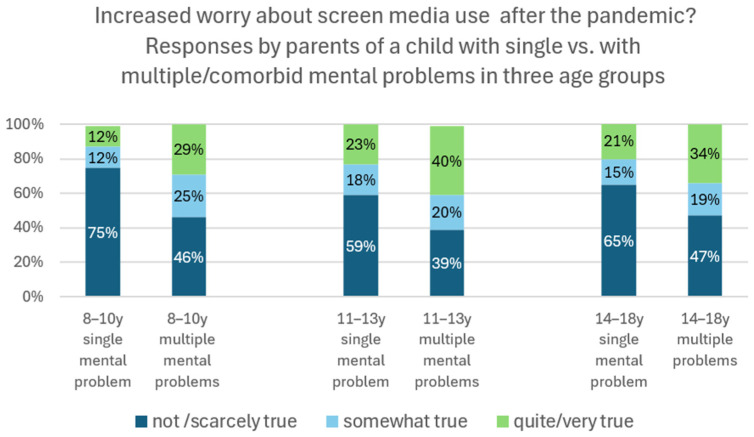
“Are you more worried today than before the pandemic about your child’s screen media use?” Responses (%) by parents of a child with a single (N = 395) and with secondary/multiple mental health problems (N = 291) in three age groups. Example: Of all parents of a child aged 14 to 18 years with multiple mental health problems (=100%), 47% did not or scarcely agreed with the statement of being more worried today about their child’s media than before the pandemic (not/scarcely true), 19% somewhat agreed (somewhat true), and 34% quite or absolutely agreed (quite/very true).

Parents of a child with secondary problems/comorbidities reported significantly more often being more worried about their child’s media use after the pandemic than before. This association between comorbidity and increased worry about screen media use as an effect of the pandemic was significant in all age groups (8–10 y: chi^2^ 11.387, *p* < 0.003; 11–13 ys: chi^2^ 7.7490, *p* < 0.29; 14–18 y: chi^2^ = 11.498, *p* < 0.001) (Figure 6).

Parents were asked if there had been time periods during the pandemic where the media consumption of their child was particularly high. Most parents (53%) opted for the lockdown as the period with the highest media use, followed by winter 2021 (46%) and autumn 2020 (34%). A proportion of 16% indicated summer 2020 and 14% indicated the last 2 weeks (=spring 2021) as periods with particularly high media use. A proportion of 16% reported that their child’s media use has never been high (Table 6).

**Table 6:** Percentage of patients feeling quite/highly burdened, with particularly high screen media use and particularly severe symptoms of the main mental health problem in different periods over the first year of the pandemic (multiple selections of periods possible).

	Feeling Quite/Heavily Burdened	Particularly High Media Use	Particularly High Symptom Severity
Lockdown	48.9%	53%	26.5%
Summer 2020	25.5%	16%	13.3%
Autumn 2020 (second wave)	42.3%	34%	18.4%
Winter 2021	40.2%	46%	16.9%
Last 2 weeks (Spring 2021)	18.3%	14%	4.4%
Never high/unchanged	-	16%	31.2%

**Figure 7:**
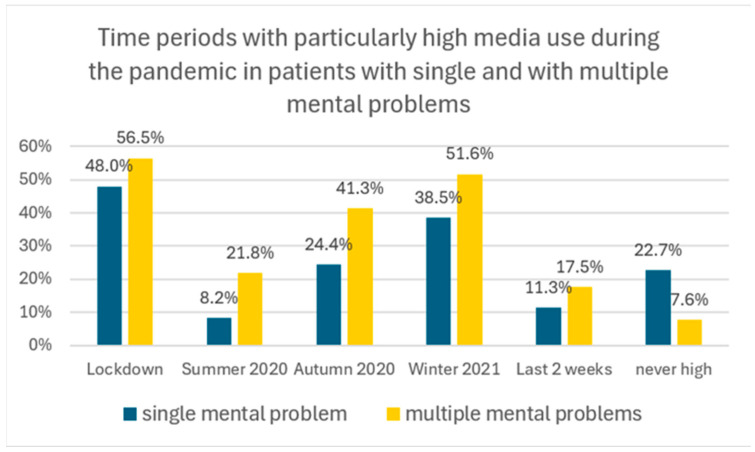
“At what times during the pandemic was your child’s screen media consumption particularly high?” Responses (%) from parents of a child with single mental problems vs. with multiple mental problems. Multiple responses were allowed. Example: 48.8% of parents of a child with a single mental problem stated that the lockdown period was associated with particularly high media use, in contrast to 56.5% of parents of a child with multiple mental health problems.

At all time periods of the pandemic, fewer patients with single mental problems showed increased screen media use compared to patients with multiple mental health problems. However, the general profile of times with intense media use—with a maximum during the lockdown—was similar in patients with single compared to patients with multiple mental health problems, albeit with less pronounced peaks in autumn 2020 and winter 2021 (Figure 7). Males and females did not differ regarding periods of increased screen media use.

### 3.8. Looking Back After the First Year of the COVID-19 Crisis

**Figure 8:**
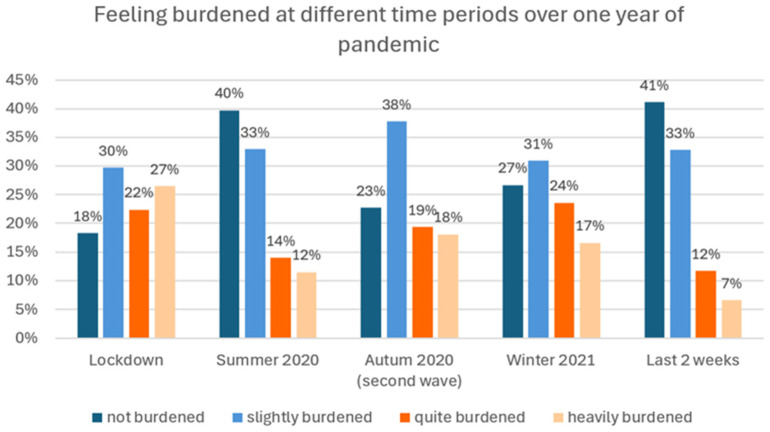
“At which time period did your child feel particularly burdened?” (% of responses; multiple responses allowed). Example: 22.7% of parents stated that their child did not feel burdened in autumn 2020, 37.8% found their child slightly burdened, 19.4% quite burdened, and 18.1% heavily burdened.

Parents were asked to estimate which time periods over the last year had been particularly difficult for their child (“When you look back at the first year of the pandemic, at which time periods did your child feel particularly burdened?”) (Figure 8). Retrospectively, the time period of the lockdown (spring 2020) was indicated by almost half of respondents as particularly difficult for their child (48.9% quite heavily burdened), followed by winter 2021 (40.2%) and autumn 2020 (“second wave”) (37.5%). Currently (the last two weeks), 18.3% continued to feel particularly burdened. Among this latter group (=100%), patients with depression (49.2%) and acute crises (23.3%) were overrepresented. At all times, significantly more parents of a child with multiple than of single disorders reported a severe psychological burden.

Similarly, parents were asked to estimate in which time periods of the first year of the pandemic that symptoms of the main mental health problem had been particularly severe. While almost one third (31.2%) indicated no relevant change throughout the year, and thus no impact of the pandemic on their child’s primary mental health condition, 26.5% estimated the lockdown period, 18.4% indicated autumn 2020, and 16.9% indicated winter 2021 as the periods with the most severe mental health symptoms.

**Figure 9:**
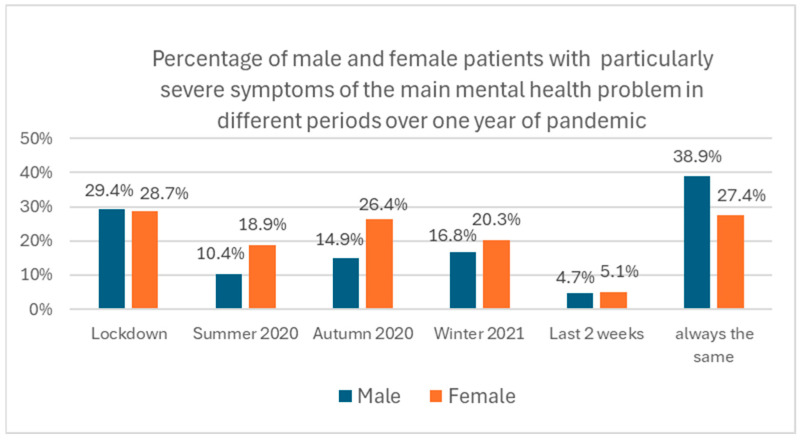
Percentage of male and female patients with particularly severe symptoms of the main mental health problem in different periods over the first year of the pandemic (multiple selections possible). Example: 14.9% of parents with a male child indicated that symptoms of the main problem had been particularly severe in autumn 2020, in contrast to 26.4% of parents with a female child.

More parents of male (38.9%) than of female (27.4%) patients reported no change in symptom severity during the first year of the pandemic (Figure 9). While the same proportion of male and female patients had the most severe symptoms during the lockdown (male, 29.4%; female, 28.7%), a high symptom severity during summer 2020 was observed more frequently in female patients. In autumn 2020, symptom severity in females approached lockdown levels. In male patients, by contrast, symptom severity never reached lockdown levels again during the later periods of the pandemic (Figure 9).

### 3.9. The Most Serious Burdens from a Parent’s Perspective

**Figure 10:**
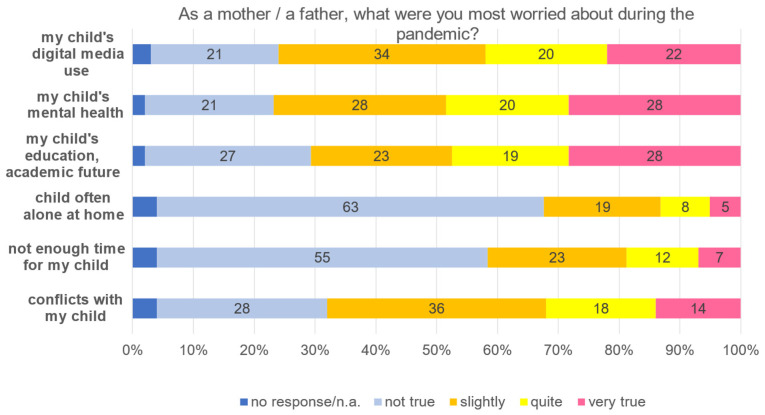
Most serious burden during the pandemic as a parent.

When asked to indicate the most severe burdens experienced during the pandemic from the perspective of a parent, almost half of the participants answered to have been worried about their child’s mental health (quite/very true 48%), followed by the worry about their child’s academic future (quite/very true 47%) and about their child’s digital media use (quite/very true 42%) (Figure 10).

**Figure 11:**
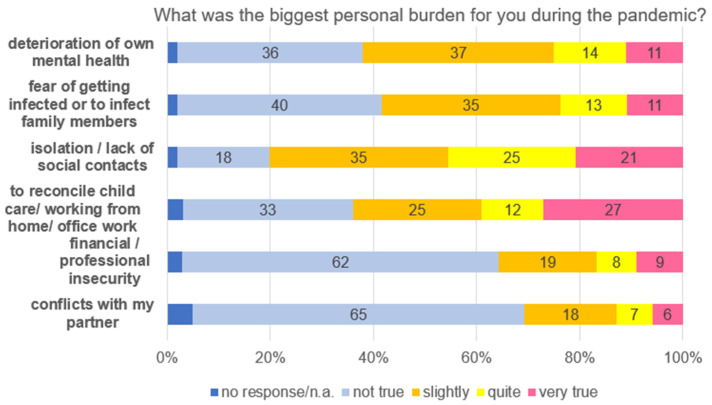
Parents’ most serious personal burden during the pandemic.

Parents were also asked for the most serious personal burdens experienced during the first year of the pandemic (Figure 11). Almost half of the participants indicated isolation and the lack of social contact (quite/very true 46%), followed by the difficulty in reconciling childcare and their job (work at home or in the office) (quite/very true 39%), and the deterioration of one’s own mental health (quite/very true 25%). Financial/professional problems and problems in the partnership were preoccupations for smaller numbers of parents (quite/very true, 17 and 13%) (Figure 11).

## 4. Discussion

We report the results of a survey on the effects of the first year of the COVID-19 pandemic conducted with parents of a child referred to child and adolescent psychiatry.

### 4.1. Impact of the Pandemic on Mental Problems

More female than male patients, especially girls aged 14 to 18 years, showed an aggravation of the main mental health problem during the pandemic, which is in line with other reports on girls being affected stronger by the pandemic than boys [34,71,73]. However, this contrasts with our first survey conducted directly after the lockdown [18], where girls had a stronger tendency for symptom improvement than boys. This suggests two possible explanations: First, female patients appear to be particularly struggling to cope with stresses that persist over longer periods of time, while males seem to adapt to the situation, and, secondly, parents need more time to accurately perceive changes in girls’ well-being who often suffer from internalizing disorders compared to boys who rather display externalizing behavioral problems.

Certain disorders—depression, acute crises, eating disorders, or obsessive-compulsive disorders—were more likely to worsen than others, e.g., ADHD or ASD. This overlaps in part with the characteristic gender distribution in child and adolescent psychiatry, with a male predominance in ADHD and ASD and a female predominance in depression (in teenagers) and in eating disorders (e.g., [74]). Another aspect is that external factors related to the pandemic, such as contact restrictions or other protective measures, probably had a stronger negative impact on some specific pre-existing symptoms and disorders (e.g., physical isolation on depression, hygiene regulations on compulsions) [33,75,76]. In some patients with ASD or anxiety disorder, by contrast, reduced physical contacts may have been experienced as a relief rather than a burden [49,50]. In addition, we found that secondary mental problems, except learning disorders, more frequently showed a worsening than the primary ones did. It is plausible that a comorbid problem was more likely to be influenced or triggered by external factors than an underlying chronic mental health condition. Specific comorbid problems like PUI or school absenteeism were probably exacerbated by the pandemic or resulted directly from the protective measures in vulnerable patients [63,64,77].

### 4.2. Screen Media Use and Its Relation to Mental Health During the Pandemic

We found a mean leisure screen media time in spring 2021 of 5.8 h per day in the oldest group (14 to 18 y), which is comparable to the mean screen media time directly after the lockdown from our first study [18]. The differences between boys and girls at that time evened out one year later (14–18 y: spring 2020 survey: females, 5.4 h; males, 6.4 h; 2021 survey: females, 5.7 h; males, 5.9 h). This mean screen media time is roughly in line with the results of a recent representative survey with Swiss youth [78]. As in our first study [18], boys spent more time video gaming than on social media, while girls spent considerably more time on social media than video gaming.

An important finding in our clinical sample was that higher total screen media time and screen media use in general were associated with multiple/comorbid psychopathological problems. Patients with secondary/comorbid problems had significantly higher screen media times, which increased overproportionally in periods of greater mental burden, compared to patients with a single problem. Similarly, a significantly higher proportion of parents of a child with multiple than with single mental health problems reported being more concerned about their child’s media consumption after the pandemic than before. This study thus indicates that the pandemic had a greater impact on media consumption among children and adolescents with more complex disorders, in a way that was perceived by parents as negative and harmful. Among the 30% of respondents who explicitly assumed a negative influence of media use on their child’s mental health, parents of a child with depression, acute crises, and eating disorders were overrepresented. Parents of a child with depression and acute crises were also particularly concerned about the high level of social media use, and parents of a child with aggression/CD, and to a lesser extent parents of a child with ADHD, were concerned about video gaming. In adolescents, associations between problematic social media use and depression, suicidal crises, or eating disorders [63,71,79,80] and between problematic video gaming and externalizing disorders such as ADHD or behavioral disorders [81,82] have also been described in the literature. Despite the high number of parents who were highly concerned about their child’s media consumption, PUI was not reported as a primary reason for referral and was indicated as a secondary problem by only N = 32 parents (<5%). This is consistent with the literature, according to which pathological screen media consumption in children and adolescents often occurs in association with other psychopathological disorders (e.g., [83]). Most parents clearly do not regard PUI as an independent or comorbid disorder, but as part of a more complex problematic behavioral or psychopathological pattern. In addition, specialized centers exist in the area for the diagnosis and treatment of “pure” PUI, while only more complex and severe cases are referred to child and adolescent psychiatry.

To conclude, when it comes to the long-term negative influence of the pandemic on media behavior, it is necessary to differentiate the following: children/adolescents with less complex/less severe disorders seemed to be able to better regulate screen media behavior and relapsed less during difficult time periods than patients with multiple disorders. A general association between pandemic peak periods, heightened emotional distress, and screen media time has also been described in adolescents from a community sample [70] and in community as well as clinical samples [43]. Interestingly, in our first survey on the impact of the pandemic on media use conducted directly after the lockdown (spring 2020), we did not observe this association between total screen media time and comorbidity [18]. According to a meta-analysis by Dal-Pai and colleagues [84], increased media time in the medium range (i.e., below 3.5 h) was associated with coping with COVID-related stress in ADHD, and over 3.5 h of use was associated with depression. Clearly, the lockdown, an unprecedented event with a massive impact on everyone’s life over a short period of time, had similar effects on most patients, while permanent stress and uncertainty in the following months were more difficult to cope with for more severely affected and more vulnerable patients. Female adolescents aged 14 to 18 years seemed to be particularly vulnerable and susceptible to harmful media behavior when under continuous stress.

### 4.3. Retrospective View on Time Periods and Personal Burdens

Most parents indicated the lockdown period as particularly burdening for their child, followed, to a lesser extent, by the “second wave” of disease in autumn 2020 and the winter months of 2021. For the majority, i.e., over 80%, the situation seemed to have returned to normal in spring 2021, but a sizable number of patients, mostly with depression and in acute crises, continued to experience major psychological burdens. For almost half of the respondents, dysregulated media use was retrospectively a major concern during the pandemic and continued to be perceived as a danger for their child’s mental health. While symptom severity decreased in most males after the lockdown, it remained at a higher level in girls throughout the year of the pandemic. Female patients clearly responded more strongly to the continued stresses that followed during the difficult months after the lockdown than male patients. In females, peaks of high media use seemed to reflect periods of high mental burden more than in male patients. The dramatic increase in demand for treatment for children and adolescents, reported by mental health professionals [39,41], particularly in winter 2021, was only partially reflected by the present data, which identified the period of major mental burden as during the lockdown, at least for the majority of patients. This may partly be explained by the fact that, as consequence of increased demands, many patients who had become mentally ill during the pandemic had to wait several months before starting outpatient treatment and thus were not included in the present sample. Only 7% of respondents reported newly emerged mental problems. According to mental health professionals, however, most of the patients with an urgent need for treatment in winter 2021 were teenage girls suffering from depression or adolescents with acute mental health crises [40], which supports our current findings.

## 5. Strengths and Limitations

In this study, we were able to show that child and adolescent psychiatric patients with multiple/comorbid mental disorders spend more time using screen media than those with single disorders, indicating an association between the complexity of mental illness and problematic media use. The identification of patients at risk is one of the objectives of current research into problematic internet use [67]; the results presented here can contribute to that aim.

There are also some limiting factors: All answers were given retrospectively, i.e., between 12 May and 4 June 2021, and may be biased accordingly. The relatively low response rate suggests that the sample may not be representative regarding SES, with the majority of respondents not affected by the negative economic consequences of the pandemic. This is, however, a characteristic bias in anonymous and voluntary surveys. Parents with immigrant backgrounds may have refrained from answering the questionnaire due to language problems. In addition, with over 90% of female responders, it is a mothers’ rather than a parents’ survey. Disorder classification was based on parental information and therefore was usually referred to as a “problem”. It must also be assumed that parents’ perceptions of their child’s media use may be distorted.

## 6. Conclusions

The present findings revealed that not only lockdown, but also the subsequent months of the pandemic with its various virus waves, had strong adverse effects on mental well-being and pre-existing psychopathology in young patients with psychiatric disorders. Although protective measures in Switzerland were less strict than in other countries and most schools remained open after the spring 2020 lockdown, the consequences of a long-lasting pandemic were still difficult to cope with for the vulnerable groups. These vulnerable patients, i.e., those with complex mental problems and teenage girls, in particular, became more vulnerable during the pandemic. From the perspective of most parents, screen media use has contributed to this development.

At the moment, it looks as if the COVID pandemic has faded into the background as the world has been in a multi-crisis over recent years. Due to new political, economic, social, and ecological changes such as wars, inflation, and the climate crisis, it seems as if the focus has shifted to new areas. This makes it even more important for people who work with children and adolescents to recognize their stress in good time and to provide them with appropriate professional support. Large-scale mental health screenings in schools and early intervention might contribute to an enhancement in mental health. Intervention programs on cognitive restructuring [85], adaptive coping strategies, and stress management and their integration into educational, clinical, and family settings may be helpful for the promotion of long-term psychological mental well-being. In addition, media education addressing the risks associated with the internet for one’s mental health and the principles of digital well-being [86,87] should be central topics in schools.

## Figures and Tables

**Table 1:** Distribution of age, gender, and frequency of comorbidity (y = years).

	N	%	Age Mean (SD)		Patients with a Secondary Problem (%)
**Male**	**342**	**49.9%**	**13.1**	**(2.85)**	**56.4%**
Male 8–10 y	83	12.1%	9.4	(0.76)	47.0%
Male 11–13 y	105	15.3%	12.0	(0.80)	55.2%
Male 14–18 y	154	22.4%	15.8	(1.26)	62.3%
**Female**	**321**	**46.8%**	**13.9**	**(2.70)**	**57.9%**
Female 8–10 y	44	6.4%	9.1	(0.76)	38.6%
Female 11–13 y	80	11.7%	12.0	(0.83)	56.2%
Female 14–18 y	197	28.7%	15.8	(1.16)	62.9%
**Other/diverse**	**23**	**3.4%**	**15.2**	**(2.6)**	**69.6%**
**All**	**686**	**100%**	**13.5**	**(2.81)**	**58.0%**
8–10 y	129	18.8%	9.3	(0.78)	43.4%
11–13 y	186	27.1%	12.0	(0.83)	55.9%
14–18 y	371	54.1%	15.8	(1.20)	53.3%

**Table 2:** Improvement and deterioration of the main mental health problem in male, female, and diverse patients, and in different age groups over the first year of the pandemic (n.a. = not applicable; y = years).

	Main Mental Health Problem over First Year of Pandemic			
	Deteriorated	Unchanged	Improved	No Answer/n.a.
**Male (N = 342)**	**22.8%**	**38.0%**	**22.3%**	**17%**
8–10 y (N = 83)	18.1%	34.9%	28.9%	18%
11–13 y (N = 105)	21.9%	42.9%	19.0%	16.2%
14–18 y (N = 154)	26.0%	36.4%	20.8%	17.8%
**Female (N = 321)**	**34.9%**	**22.7%**	**18.4%**	**23.6%**
8–10 y (N = 44)	22.7%	31.8%	29.5%	15.9%
11–13 y (N = 80)	35.0%	27.5%	18.8%	18.8%
14–18 y (N = 197)	37.6%	18.8%	15.7%	28%
**Gender dysphoria (** **N = 23)**	**21.7%**	**34.8%**	**21.7%**	**21.7%**
**All (N = 686)**	**28.4%**	**30.8%**	**20.4%**	**20.4%**

**Table 3:** Changes in symptom severity of the main and secondary mental problems during the pandemic (only valid responses included (=100%)).

Main Mental Problem		Change in Severity During Pandemic		
	**N**	**Worse**	**Same**	**Improved**
ADHD	162	28.4%	47.5%	24.1%
Autism spectrum disorder	79	29.1%	48.1%	22.8%
Depression	55	58.2%	18.2%	23.6%
Anxiety	52	28.8%	34.6%	36.5%
OCD	32	46.9%	25.0%	28.1%
Acute crisis	23	56.5%	17.4%	26.1%
Gender dysphoria	18	27.8%	44.4%	27.8%
Eating disorder	14	57.1%	28.6%	14.3%
Aggression/CD	21	33.3%	23.8%	42.9%
Other	66	31.8%	42.1%	25.8%
Not specified	24	41.7%	45.8%	12.5%
**All**	**546**	**37.7%**	**38.6%**	**25.6%**
**Secondary mental problem**		**Change in severity during pandemic**		
	**N**	**Worse**	**Same**	**Improved**
ADHD	51	41.2%	51.0%	7.8%
Anxiety	64	46.9%	32.8%	20.3%
Depression	73	50.7%	26.0%	23.3%
Absenteeism/withdrawal	37	67.6%	21.6%	10.8%
OCD	37	40.5%	40.5%	18.9%
Learning disorder	40	15.0%	60.0%	25.0%
PUI	25	76.0%	20.0%	4.0%
Eating disorder	16	68.8%	18.8%	12.5%
Other	23	52.2%	43.5%	4.3%
**All**	**366**	**48.1%**	**35.8%**	**16.1%**

**Table 4:** Estimated total screen media time and time spent on different devices in male, female, and diverse patients and different age groups (y = years).

		Mobile	PC/Tablet	Gaming Console	TV	Total Screen Media Time
	N	Time (h) Mean *(SD)*	Time (h) Mean *(SD)*	Time (h) Mean *(SD)*	Time (h) Mean *(SD)*	Time (h) Mean *(SD)*
**8–10 y all**	129	0.66 *(1.10)*	0.82 *(1.11)*	0.37 *(0.65)*	0.74 *(0.88)*	**2.58 *(2.12)***
Boys 8–10 y	83	0.74 (*1.24)*	0.83 *(1.19)*	0.45 *(0.70)*	0.78 *(0.72)*	**2.80 *(2.36)***
Girls 8–10 y	44	0.52 *(0.78)*	0.80 *(0.94)*	0.24 *(0.52)*	0.68 *(0.70)*	**2.25 *(1.58)***
**11–13 y all**	186	1.76 (*1.80)*	1.37 *(1.49)*	0.61 *(1.28)*	0.79 *(1.0)*	**4.52 *(3.59)***
Male 11–13 y	105	1.56 *(1.73)*	1.40 *(1.51)*	0.86 *(1.41)*	0.79 *(1.15)*	**4.62 *(3.83)***
Female 11–13 y	80	2.0 *(1.87)*	1.26 *(1.46)*	0.27 *(0.88)*	0.78 *(0.78)*	**4.33 *(3.23)***
**14–18 y all**	371	3.23 *(2.07)*	1.54 *(1.68)*	0.45 *(0.95)*	0.60 *(0.86)*	**5.83 *(3.38)***
Male 14–18 y	157	2.84 *(1.97)*	1.79 *(1.94)*	0.76 *(1.13)*	0.53 *(0.72)*	**5.94 *(3.40)***
Female 14–18 y	197	3.49 *(2.11)*	1.35 *(1.42)*	0.20 *(0.70)*	0.65 *(0.96)*	**5.70 *(3.39)***
**All**	686	2.35 *(2.10)*	1.36 *(1.55)*	0.48 *(1.01)*	0.68 *(0.88)*	**4.81 *(3.46)***
Male all	342	1.94 *(1.94)*	1.44 *(1.69)*	0.72 *(1.17)*	0.67 *(0.88)*	**4.77 *(3.54)***
Female all	321	2.71 *(2.20)*	1.26 *(1.38)*	0.23 *(0.73)*	0.69 *(0.88)*	**4.88 *(3.37)***
Diverse	23	3.41 *(2.04)*	1.54 *(1.83)*	0.52 *(1.13)*	0.58 *(1.11)*	**6.06 *(2.12)***

**Table 5:** Parents’ concern about their child’s screen media use and its negative impact on everyday life.

**Worry About Screen Media Time** *I Am Worried About the Amount of Time Spent by My Child…*	**Percent of Responses (%)**			
	**Not True**	**Slightly True**	**Quite True**	**Absolutely True**
… on his/her smartphone	23.0	32.2	29.2	15.6
… on the internet	21.4	37.5	27.0	14.1
… playing videogames	41.1	29.7	16.3	16.3
… on social networks	38.9	29.6	17.8	13.7
… watching TV	65.0	24.5	8.0	2.5
**Negative Impact on Everyday Life** *Negative impact of my child’s media use on…*	**Not True**	**Slightly True**	**Quite True**	**Absolutely True**
… family life	24.6	40.4	23.8	11.2
… homework and academic achievements	38.9	37.3	16.6	7.1
… friendships and social activities in real life	42.9	34.3	16.2	6.7
… mental well-being and mental health (e.g., mood)	32.4	38.0	21.7	7.9
… physical well-being and health (e.g., sleep)	35.7	38.8	17.8	7.7
*I am concerned because my child …*				
… becomes aggressive/very angry when media use is restricted	33.7	30.2	20.3	15.9
… secretly spends more time on media than agreed upon ^(a)^	35.3	28.3	14.7	13.3
**Risky/problem behaviors** *I am concerned that my child might…*	**Not True**	**Slightly True**	**Quite True**	**Absolutely True**
… be a victim of cyberbullying	35.3	28.3	10.6	5.8
… be a cyberbullying perpetrator	76.8	16.6	3.6	2.9
… play video games with harmful or age-inappropriate content (e.g., trivializing violence)	54.4	30.2	10.2	5.2
… watch films, series or clips with harmful or age-inappropriate content	34.3	39.5	18.4	7.9

Remark. N = 686; (a) 8.5% had no agreement with parents/not applicable.

## Data Availability

The original contributions presented in this study are included in the article/Supplementary Materials. Further inquiries can be directed to the corresponding author(s).

## Supplementary Materials

The following supporting information can be downloaded at: https://www.mdpi.com/article/10.3390/healthcare13162026/s1, Table S1. Chronology of protective measures during the COVID-19 Pandemic in Switzerland. Table S2. Distribution of main and secondary psychopathological problems. Table S3. Screen mean media time on different devices and total media time in male and female patient groups at different ages (non-users included). Table S4. Estimated screen mean media time (hours, SD) on different devices in users only, in male, female and diverse patients and different age groups. Table S5. Estimated video gaming time and social media time (hours, SD) in male and female patients, users only, in different age groups. Table S6. Estimated screen mean total media time in patients with single mental problem and multiple problems in different age groups. Figure S1. “Are you more worried today than before the pandemic about your child’s screen media use?” Responses (%) by parents of male (N = 342) and female (N = 321) patients.
