# Supplementary material for: Looking Back After the First Year of the COVID-19 Pandemic: Parents’ View on Screen Media Use, Psychopathology, and Psychological Burden in a Clinical Sample of Children and Adolescents
Source: Healthcare (Basel). 2025 Aug 17;13(16):2026. doi: 10.3390/healthcare13162026 (PMC12385759; doi:10.3390/healthcare13162026)
Supplement: Supplementary file 1 [file healthcare-13-02026-s001.zip › healthcare-3757890-supplementary.pdf]

Supplementary Materials

Anna Maria Werling, Susanne Walitza, and Renate Drechsler

Looking back after the first year of the COVID-19 Pandemic: A parents' view on screen media use, psychopathology, and psychological burden in a clinical sample of children and adolescents

Healthcare, 2025

Table S1. Chronology of protective measures during the COVID-19 pandemic in Switzerland.

**Spring 2020 "Lockdown"**	**Complete lockdown with school closure, 16 March to the end of April 2020. First schools reopened in May 2020.**
**Summer 2020 (June–September)**	Most societal measures lifted; the minimum distance reduced to 1.5 meters. Borders for several countries reopened. Most schools open.
**Autumn 2020 "Second wave" (October–December)**	October: No gatherings of more than 15 people in public spaces. Discotheques and dance halls closed; curfew from 11 pm to 6 am. Indoor sports and cultural leisure activities allowed up to 15 people with physical distance and masks. November: Switch to distance learning in universities. "Onsite" teaching remained permitted in compulsory and secondary schools. 19 December: Complete closure of restaurants, sport, and cultural facilities. Recommendation to stay at home.
**Winter 2021 (January–March)**	Closure of restaurants, cultural, sports, and leisure facilities until the end of February. First vaccines available for high-risk patients. 1 March: Gradual reopening of stores, museums, sport, cultural, and leisure facilities with masks, social distancing, and capacity restrictions. From 22 March onwards, cultural and sporting events with an audience within a strictly limited framework.
**Spring 2021 "Last two weeks" (with reference to data collection 12 May to 4 June)**	Gradual reopening. Vaccines available for the general population. End of May: Reopening of indoor spaces in restaurants and other facilities with safety measures and collection of contact data.

Table S2. Distribution of main and secondary psychopathological problems.

**Main problem**	**N**	**Sex m/f**	**Age mean *(SD)* (y)**	**Secondary problem/comorbidity (N)**									
				None	OCD	Abs	ADHD	Dep	PUI	Anx	Eat	LD	oth
ADHD	176	123/53	12.62 *(2.76)*	86	6	8	0	10	15	16	1	20	14
ASD	90	66/24	13.88 *(2.87)*	31	10	2	22	6	4	7	1	5	2
Dep	74	22/52	15.14 *(2.15)*	16	2	13	6	1	1	21	9	2	3
Anx	*65*	*24/41*	*13.05 (3.04)*	33	6	7	2	10	0	1	0	5	2
OCD	37	14/23	14.38 (2.87)	15	1	1	3	4	3	9	0	1	0
Crisis	40	10/30	15.38 *(1.69)*	5	2	1	0	21	0	6	2	0	2
Gender D	23		15.71 *(2.59)*	7	2	2	1	9	0	1	0	0	1
Eat	*31*	2/29	*15.26 (1.34)*	15	5	1	0	8	0	1	0	0	1
CD	*25*	*17/8*	*12.44 (2.77)*	9	0	1	4	2	2	1	1	4	1
Assess	26	*17/9*	11.19 *(2.31)*	19	1	1	1	1	2	0	0	0	1
LD	12	6/6	12.02 *(4.69)*	8	0	0	2	0	0	0	0	1	1
Tic	16	13/3	13.13 *(2.58)*	12	1	1	1	0	0	1	0	0	0
Trauma	6	1/5	15.67 *(0.66)*	4	0	0	0	2	0	0	0	0	0
Social	7	2/5	12.29 *(3.04)*	5	0	0	0	1	0	0	0	0	1
Psychsom	5	2/3	13.00 *(1.41)*	5	0	0	0	0	0	0	0	0	0
Psych	3	1/2	14.33 *(0.57)*	1	1	0	0	0	0	0	0	1	0
Abs	3	1/2	8.67 *(0.57)*	3	0	0	0	0	0	0	0	0	0
Other	13	9/5	13.00 *(3.02)*	4	0	1	0	2	4	0	0	0	2
Not spec	34	9/16	13.62 *(2.41)*	13	1	1	3	7	1	4	3	1	1

Abs = school absenteeism/social withdrawal/mutism; ADH = attention deficit hyperactivity disorder; Anx= anxiety; ASD = autism spectrum disorder; Assess = assessment (reason for referral); CD = conduct disorder/aggressiveness; Crisis = acute crisis/suicidality; Dep = depression; Eat = eating disorder; Gender D = gender dysphoria; LD = learning disorder (dyslexia; dyscalculia); OCD = obsessive–compulsive disorder; Tic = tic disorder; Social = social/family problems; Psychsom = psychosomatic problems; Psych = psychosis/borderline; PUI = problematic use of the internet/gaming; Oth = other; Not spec = not specified. y = years.

Table S3. Screen mean media time on different devices and total media time in male and female patient groups at different ages (non-users included).

	N	Mean time (hours) spent on Mobile	PC/ tablet	Gaming console	TV	Total screen media time Mean	Total screen media time SD
**8-10 y all**	**129**	**.66**	**0.82**	**.37**	**.74**	**2.58**	**(2.12)**
Male	83	.74	0.83	.45	.78	2.80	(2.36)
Female	44	.52	0.80	.24	.68	2.25	(1.58)
**11-13 y all**	**186**	**1.76**	**1.37**	**.61**	**.79**	**4.52**	**(3.59)**
Male	105	1.56	1.40	.86	.79	4.62	(3,83)
Female	80	2.0	1.26	.27	.78	4.33	(3.23)
**14-18 y all**	**371**	**3.23**	**1.54**	**.45**	**.60**	**5.83**	**(3.38)**
Male	157	2.84	1.79	.76	.53	5.94	(3.40)
Female	197	3.49	1.35	.20	.65	5.70	(3.39)
**Total mean**	**686**	**2.35**	**1.36**	**.48**	**.68**	**4.81**	**(3.46)**
Male	342	1.94	1.44	.72	.67	4.77	(3.54)
Female	321	2.71	1.26	.23	.69	4.88	(3.37)
Diverse	23	3.41	1.54	.52		6.06	(3.44)

SD = standard deviation. y = years

Table S4. Estimated screen mean media time (hours, SD) on different devices in users only, in male, female, and diverse patients and different age groups.

	**Mobile**		**PC/tablet**		**Gaming console**		**TV**	
	Time (hrs) mean (SD)	% user	Time (hrs) mean (SD)	% user	Time (hrs) mean (SD)	% user	Time (hrs) mean (SD)	% user
**8-10 yrs all**	**1.36 (1.23)**	**48.8**	**1.13 (1.16)**	**72.1**	**1.00 (.71)**	**37.7**	.92 (.67)	**77.5**
Boys 8-10 y	1.42 (1.40)	50.6	1.21 (1.27)	68.7	1.04 (.73)	43.4	.95 (.69)	80.7
Girls 8-10 y	1.21 (0.76)	43.2	1.01 (.95)	79.5	.87 (.67)	27.8	.90 (.97)	75.0
**11-13 y all**	**2.04 (1.78)**	**86**	**1.61(1.55)**	**84.9**	**1.48 (1.64)**	**40.9**	**1.12 (1.03)**	**69.9**
Male 11-13 y	1.86 (1.73)	83.8	1.67(1.51)	83.8	1.61 (1.68)	53.3	1.17(1.23)	67.6
Female 11-13 y	2.26 (1.83)	88.7	1.46 (1.48)	86.2	1.15(1.54)	23.7	1.06 (.73)	73.6
**14-18 y all**	**3.28 (2.04)**	**98.7**	**1.82 (1.66)**	**84.4**	**1.42 (1.25)**	**32.1**	**1.08 (0.94)**	**56.1**
Male 14-18 y	2.89 (1.94)	98.1	2.09 (1.27)	85.7	1.54 (1.17)	49.2	1.01 (.71)	53.3
Female 14-18 y	3.95 (2.09)	99.0	1.63 (1.40)	83.2	1.14 (1.32)	17.8	1.11 (1.35)	58.9
**All**	**2.73 (2.03)**	**85.9**	**1.65 (1.57)**	**82.2**	**1.35 (1.13)**	**35.6**	**1.05 (.92)**	**64.4**
Male all	2.36 (1.90)	84.2	1.78 (1.71)	81.0	1.46 (1.31)	49.1	1.04 (.91)	64.3
Female all	3.05 (2.04)	88.8	1.50 (1.38)	83.5	1.09 (1.29)	20.6	1.06 (.90)	64.8
Diverse	3.41 (2.04)	100	1.86 (1.45)	82.6	1.33 (1.52)	39.1	1.12 (1.35)	52.2

Table S5. Estimated video gaming time and social media time (hours, SD) in male and female patients, users only, in different age groups.

	Video Gaming			Social media		
	Gaming time (hrs) Mean (SD)	Gaming user N	Male vs. female[1]	Social media time (h) Mean (SD)	Social media users N	Male vs. female[1] p
8-10 y all	1.13 (1.10)	89		1.09 (1.36)	26	
Boys	1.23 (1.20)	68	M>F(*)	1.23 (1.43)	19	ns
Girls	.78 (.60)	21		0.71 (0.56)	7	
11-13 y all	1.64 (1.60)	125		1.71 (1.51)	131	
Male	1.80 (1.62)	88	ns	1.51 (1.43)	62	ns
Female	1.26 (1.50)	36		1.88 (1.59)	68	
14-18 y all	2.04 (1.79)	210		2.36 (1.96)	333	
Male	2.34 (1.86)	131	M>F***	1.74 (1.68)	125	F>M***
Female	1.51 (1.59)	66		2.78 (2.06)	188	
Total	1.73 (1.10)	424		2.11 (1.86)	490	
Male all	1.91 (1.71)	287	M>F***	1.63 (1.60)	206	F>M***
Female all	1.31 (1.46)	123		2.49 (1.98)	263	
Diverse	1.67 (1.20)	14		2.14 (1.56)	21	

F = female; M = male ; *** $p<0.001$; ns = non-significant. y = years

Table S6. Estimated total mean screen media time in patients with single mental problem and multiple problems in different age groups.

		Single mental health problem		Multiple mental health problems	Post hoc t-tests
Age group	N	Total media time (hrs) Mean (SD)	N	Total media time (hrs) Mean (SD)	p
**All**	**291**	**3.97 (2.94)**	**395**	**5.53 (3.67)**	**0.001**
8-10 y	73	2.23 (1.67)	56	3.04 (2.54)	0.043
11-13 y	82	3.86 (3.38)	104	5.05 (3.66)	0.024
14-18 y	136	4.97 (2.76)	235	6.34 (3.60)	0.001

y = years; ANOVA mental health groups (2) by age groups (3): mental health group: $F=17.118$, $p<0.001$,
- Age group: $F=42.197$; $p<0.001$;
- Mental health by age group: ns.

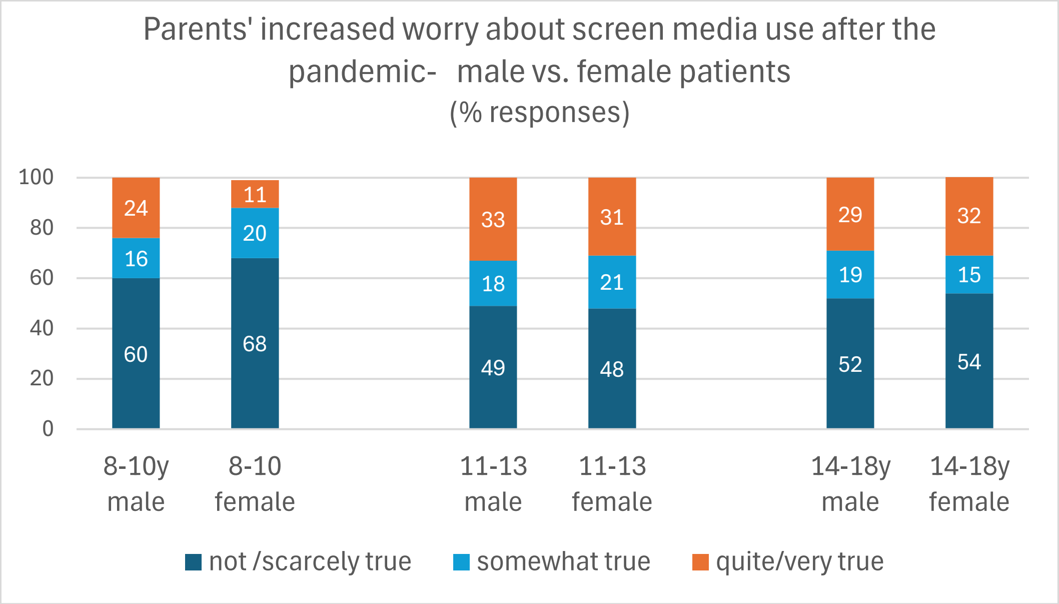

Figure S1. "Are you more worried today than before the pandemic about your child's screen media use?" Responses (%) by parents of male (N=342) and female (N=321) patients.
